# The Endothelial Glycocalyx: A Possible Therapeutic Target in Cardiovascular Disorders

**DOI:** 10.3389/fcvm.2022.897087

**Published:** 2022-05-13

**Authors:** Anastasia Milusev, Robert Rieben, Nicoletta Sorvillo

**Affiliations:** ^1^Department for BioMedical Research (DBMR), University of Bern, Bern, Switzerland; ^2^Graduate School for Cellular and Biomedical Sciences (GCB), University of Bern, Bern, Switzerland

**Keywords:** glycocalyx, endothelial cell (EC), cardiovascular risk factor, ischemia/reperfusion injury, inflammation, therapeutic target, heparan sulfate (HS), atherosclerosis

## Abstract

The physiological, anti-inflammatory, and anti-coagulant properties of endothelial cells (ECs) rely on a complex carbohydrate-rich layer covering the luminal surface of ECs, called the glycocalyx. In a range of cardiovascular disorders, glycocalyx shedding causes endothelial dysfunction and inflammation, underscoring the importance of glycocalyx preservation to avoid disease initiation and progression. In this review we discuss the physiological functions of the glycocalyx with particular focus on how loss of endothelial glycocalyx integrity is linked to cardiovascular risk factors, like hypertension, aging, diabetes and obesity, and contributes to the development of thrombo-inflammatory conditions. Finally, we consider the role of glycocalyx components in regulating inflammatory responses and discuss possible therapeutic interventions aiming at preserving or restoring the endothelial glycocalyx and therefore protecting against cardiovascular disease.

## Introduction

The endothelial glycocalyx is a carbohydrate-rich layer of proteoglycans and glycosaminoglycans lining the luminal surface of endothelial cells (ECs) in all blood vessels, ranging from small capillaries (1) to large arteries and veins (2). The thickness and structure of the glycocalyx differs between vascular beds and is highly dependent on the shear stress applied to the EC surface (3, 4). The expression of glycocalyx components is regulated and maintained by physiological blood flow conditions, leading to an intact glycocalyx layer. The thickness of the glycocalyx ranges from a few nanometers up to one micrometer. However, the observed thickness depends largely on the investigation method and whether the measurement was performed *in vivo* or on fixed tissues (5–7). Interestingly, glycocalyx thickness correlates with glycocalyx integrity and function. In fact, reduced thickness has been observed in inflammatory disorders and is associated with vessel dysfunction (8).

The endothelial glycocalyx fulfills diverse functions, ranging from mechanotransduction, maintenance of vascular integrity and vascular tone (9), to supporting the production of nitric oxide (NO) as well as providing anti-inflammatory and anti-coagulant properties by interacting with plasma proteins and plasma cells (10, 11). Two important families of proteins that interact with the glycocalyx and help maintaining vascular hemostasis are complement regulatory proteins including factor H (fH) and C1 inhibitor (12), and regulatory proteins of the coagulation system such as antithrombin III (ATIII) (13). These proteins, like many other plasma proteins, interact with the glycocalyx using heparan sulfate (HS) binding domains (14). The specific interaction of these proteins with HS underlines the importance of the structure and integrity of the glycocalyx for EC function. In fact, alterations of the glycocalyx cause loss of protein binding and are associated with disease. During several thrombo-inflammatory conditions including atherosclerosis, myocardial infarction, stroke, and ischemia/reperfusion injury, but also during infections and sepsis, syndecans (a core protein) or HS are released from the endothelial surface and represent a readout of glycocalyx damage (15–17). Once released, glycocalyx components induce activation of dendritic cells causing secretion of pro-inflammatory cytokines (18). At the endothelial level, partial or total shedding of the glycocalyx leads to increased leukocyte rolling and adhesion (19–21), elevated vessel permeability (22, 23), impaired vascular tone (24), and coagulation (8). The essential role of the glycocalyx in maintaining vascular hemostasis underscores the importance of protecting or restoring the glycocalyx as a potential therapeutic intervention for many inflammatory disorders. In this review we discuss the physiological functions of the glycocalyx with particular focus on how alteration of its integrity plays a role downstream of multiple cardiovascular risk factors in the context of thrombo-inflammatory conditions. Finally, we explore how prevention of shedding or restoration of the glycocalyx integrity can protect against the development of inflammatory disorders and therefore represent a potential therapeutic target.

## Biosynthesis And Composition Of The Endothelial Glycocalyx

The glycocalyx consists of different glycoproteins and proteoglycans. Glycoproteins contain oligosaccharide chains with terminal sialic acid residues (25) while proteoglycans are membrane-anchored core proteins covalently linked to negatively charged glycosaminoglycan (GAG) chains through tetrasaccharide bridges (26). The mechanical features of each GAG chain are determined by its specific sugar composition, a combination of disaccharides containing hexosamine (N-sulfated or N-acetylated) and uronic acid or galactose residues. This results in the formation of the five primary groups of GAGs: heparan sulfate, chondroitin sulfate, keratan sulfate, dermatan sulfate, or hyaluronan (8, 10, 26).

**Figure 1:**
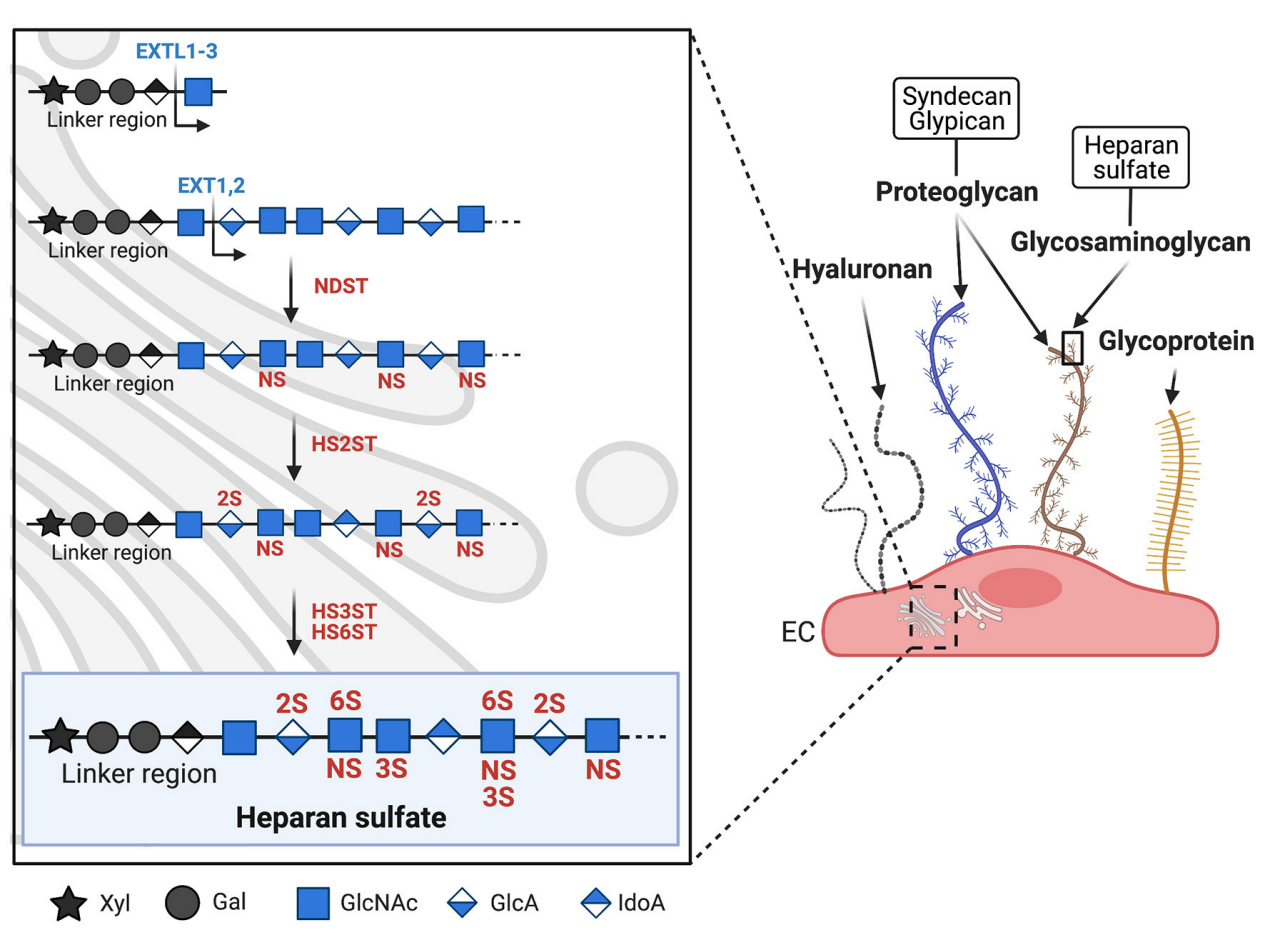
Biosynthesis and structure of the endothelial glycocalyx. Schematic representation of the major glycocalyx components covering the luminal surface of microvascular endothelial cells (EC). On the right panel, syndecan (blue) and glypican (brown) are shown as two examples of proteoglycans (PGs, see text chapter 'Biosynthesis and composition of the endothelial glycocalyx'). PGs carry long glycosaminoglycan (GAGs) side chains, while other glycoproteins (shown in yellow) carry shorter, unbranched carbohydrate side chains. The left panel shows the biosynthesis of heparan sulfate (HS), the major GAG expressed on EC. HS biosynthesis takes place in the Golgi apparatus and is mediated by different enzymes. Synthesis is initiated by the enzyme EXTL1-3 which adds the first sugar to the linker region. Chain elongation is performed by EXT1-2 which add GlcNAc and GlcA. Sulfotransferases then initiate HS sulfation, starting with NDST which sulfates GlcNAc at the N-acetyl position. HS2ST sulfates uronic acid, HS3ST and HS6ST finish sulfation by adding sulfate respectively to the 3-O and 6-O position of GlcNAc. Xyl, xylose; Gal, galactose; GlcNAc, N-acetylglucosamine; GlcA, glucuronic acid; IdoA, iduronic acid; NS, N-sulfation; 2S, 2-O sulfation; 3S, 3-O sulfation; 6S, 6-O sulfation; EXTL, exostosin-like glycosyltransferase; NDST, N-deacetylase/N-sulfotransferase; HS2ST, HS 2O-sulfotransferase; HS3ST, HS 3O-sulfotransferase; HS6ST, HS 6O-sulfotransferase.

Both the protein and polysaccharide components of PGs are synthesized in the endoplasmic reticulum and Golgi apparatus, except for hyaluronan, which in turn is synthesized directly on the cell surface (27). Once decorated with a variety of different GAGs, PGs are transported from the Golgi apparatus to the cell surface where they embed into the plasma membrane. The majority of endothelial PGs are membrane anchored syndecans and glypicans, which can be associated with all five different combinations of the above-mentioned GAGs (26). The resulting large variety of PG composition makes the glycocalyx extremely complex and variable between vessels and could therefore contribute, in addition to heterogeneity given by vessel type and organ function, to the different behavior of ECs in cardiovascular disorders. The most common GAG side chain found on ECs is HS (28), which, together with chondroitin sulfate, is the main GAG linked to syndecan. As shown in Figure 1, HS is composed of repeated disaccharide units consisting of a uronic sugar, β-D-glucuronic acid (GlcA) or α-L-iduronic acid (IdoA), and an amino sugar, N-acetyl-α-D-glucosamine (GlcNAc) or N-sulfo-α-D-glucosamine (GlcNS) (29). HS synthesis is initiated by addition of GlcNAc to a carbohydrate linker attached to the PG core protein and is mediated by the exostosin-like (EXTL1-3) family of glycosyltransferases. The growing GAG chain is then extended by a HS-polymerase complex consisting of EXT1 and EXT2 that adds the monosaccharides uronic acid or D-glucosamine (Figure 1) (30, 31). HS is highly heterogenic and undergoes several steps of post-translational modifications, in which the transfer of sulfate groups to distinct positions within the GlcNAc monosaccharide region leads to N-, 2-O-, 3-O-, or 6-O-sulfation (Figure 1). These modifications are mediated by specific sulfotransferases: N-deacetylase/N-sulfotransferase (NDST) initiates N-sulfation already during synthesis of the growing GAG chain, HS 2-O-sulfotransferase (HS2ST) sulfates uronic acid during chain elongation, HS 3-O-sulfotransferase (HS3ST) and HS 6-O-sulfotransferase (HS6ST) finalizes the sulfation of HS by adding sulfate groups to the 3-O and 6-O position within GlcNAc (Figure 1). HS sulfation motifs are instrumental to the interaction with plasma proteins; fibroblast growth factor 2, for example, requires NS- and 2O-sulfation, and ATIII specifically recognizes 3O-sulfation (31–33). Binding of these proteins to the glycocalyx is essential for their activity (12, 34).

The expression of the GAG modifying enzymes is tightly regulated and has been shown to change under inflammatory conditions. For instance, the expression of NDST1 in cultured human microvascular ECs was shown to increase after stimulation with interferon gamma (IFNγ) or tumor necrosis factor alpha (TNFα). This boosts the HS N-sulfation, leading to an enhanced interaction with the chemokine CCL5 and a rise in leukocyte adhesion (35). Elevated levels of TNFα have also been associated with a decrease in HS6ST expression. This alters the sulfation pattern of HS and consequently induces leukocyte rolling and adhesion (19). Furthermore, HS acts as a viral attachment factor, facilitating viral entry and infection. This is currently of great interest in the context of the global coronavirus disease-19 (COVID-19) pandemic, as SARS-CoV-2 requires HS as a recognition molecule for infection (36). In particular, the SARS-CoV-2 spike protein specifically binds N- and 6-O sulfated HS domains while other viruses such as herpes simplex virus require 3-O sulfation (37). Based on this, it is reasonable to speculate that changes in the sulfation pattern contribute to the different viral infection rates observed in the population. Further studies should assess whether modifications on HS play a role in EC infection.

Altogether, post-translational modifications of HS are largely responsible for changes in the interaction of plasma proteins, cells and virus with the endothelial glycocalyx. The differential regulation of HS modifying enzymes accounts for alterations in glycocalyx structure and function during pathological conditions.

## Functions Of The Endothelial Glycocalyx Under Physiological Conditions

**Figure 2:**
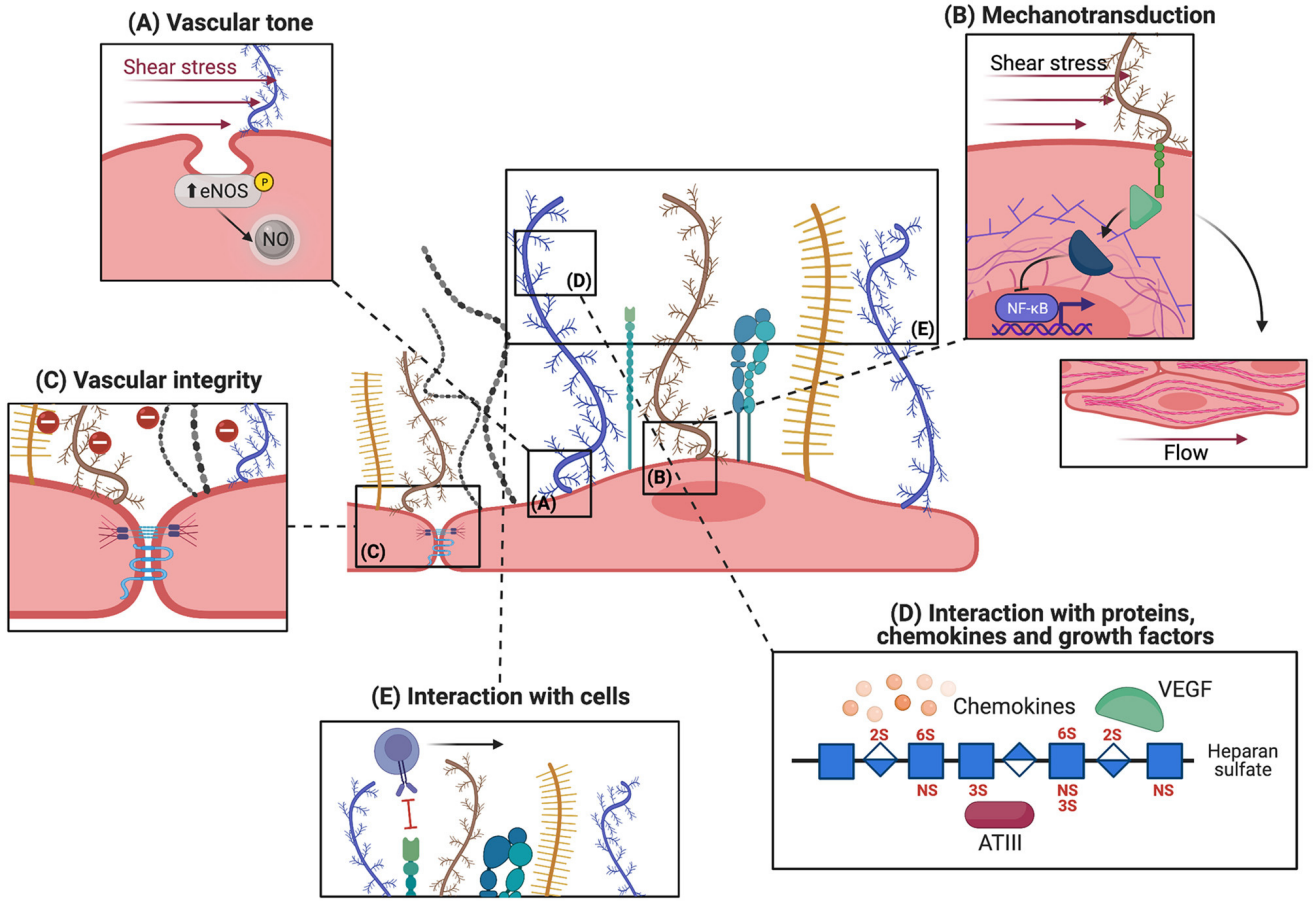
Physiological functions of the endothelial glycocalyx. The microvascular glycocalyx fulfills both mechanical and biochemical functions under physiological conditions, the five main functions are depicted here. **(A)** Sensing and transmission of shear stress by syndecans (shown in blue), located near membrane caveolae, increases eNOS activity and NO release. This assures vasodilatory functions. **(B)** Transmission of shear stress to the cytoskeleton via the cytoplasmic linker region (green) of syndecans leads to rearrangement of actin filaments and cell alignment in the direction of flow. Mechanotransduction also activates intracellular signaling molecules like Rho GTPases which regulate NF_K_B and MAPK. **(C)** The glycocalyx acts as a charge- and size-selective barrier to proteins. HS is important for maintaining intact junctions and vascular integrity. **(D)** Different molecules can bind to glycocalyx components: regulatory plasma proteins whose activity is potentiated by interaction with the glycocalyx, chemokines which are locally concentrated, and growth factors. **(E)** The intact glycocalyx shields off selectins and integrins expressed on EC thereby inhibiting leukocyte adhesion.

Initially thought to be a passive layer on the EC surface, it is now accepted that the glycocalyx serves multiple functions and actively takes part in regulating vascular hemostasis. The functions of the glycocalyx are mediated by both its mechanical and biochemical properties. Mechanical functions enclose the maintenance of vascular tone, mechanotransduction of extracellular signals, as well as preservation of vascular integrity, while biochemical functions are mediated by the interaction with plasma proteins and plasma cells (Figure 2). The vascular tone is mainly regulated by nitric oxide (NO), a vasodilator produced by ECs. When released abluminally, NO interacts with the adjacent vascular smooth muscle cells and activates soluble guanylate cyclase (sGC), which converts guanosine-triphosphate (GTP) to cyclical guanosine-monophosphate (cGMP). This leads to activation of protein kinase G and subsequently decreases intracellular calcium levels, causing relaxation of smooth muscle cells and vasodilation. A major regulator of NO production is vascular shear stress, which induces the expression of nitric oxide synthase (eNOS), the enzyme responsible for NO production within the endothelium (38, 39). On the other hand, removal of glycocalyx components has been shown to block the expression of NO and impair flow-mediated vasodilation (40–42). Consistent with this, eNOS can be upregulated only when HS structures are preserved, but not after shedding of HS by heparinase III treatment (43). Similarly, enzymatic shedding of chondroitin sulfate and sialic acids from cultured aortic ECs blocks shear-induced NO production (40). Overall, these reports indicate that the glycocalyx plays a significant role in shear-dependent NO release, but the mechanistic basis is still poorly explored. For instance, Boo et al. suggested that the increase in NO production is mediated by transmission of mechanosignals through membrane anchored PGs such as syndecan and glypican-1, located in membrane caveolae. The force applied to the glycocalyx components in the caveolae leads to phosphorylation of eNOS in a phosphoinositide-3-kinase and protein kinase A-dependent manner, thereby activating and increasing NO production (Figure 2A) (44). In line with this evidence, Ebong et al. showed that glypican-1 indeed plays a crucial role in shear-induced production of NO by phosphorylating eNOS, in such a way that silencing of glypican-1 abrogated eNOS activation (45).

The coupling of an extracellular signal, such as shear stress, to intracellular changes like expression of eNOS as detailed above, illustrates how the glycocalyx plays a significant role in mechanotransduction. Along with this concept, syndecans interact using their cytoplasmatic tail with intracellular linker proteins dynamin, tubulin and actin, and transfer shear stress signals to the cytoskeleton leading to the alignment of ECs in the direction of flow (Figure 2B) (46, 47). This alignment can be clearly visualized by the rearrangement of actin microfilaments into a series of parallel elongated fibers along the axis of ECs (48). In line with this, knock-out of syndecan-4 is associated with alignment disruption both *in vivo* and *in vitro* (49). Besides transducing shear stress information to cytoplasmic components, syndecans also impact the organization of tight junction complexes, possibly affecting vascular permeability/integrity (50). In addition, mechanotransduction of shear stress regulates a range of intracellular pathways involved in control of thrombosis and fibrinolysis, such as thrombomodulin and tissue plasminogen, and inflammation (51). For example, laminar unidirectional shear stress activates Rho family GTPases, which results in the transcriptional activity of NFκB (Figure 2B). This pathway controls the expression of the NLRP3, pro-IL-1β, and pro-IL-18 genes, which participate in inflammasome assembly (51). Similarly, shear stress controls the activation of Ras-Mitogen-Activated Protein Kinases (MAPKs) in ECs, ultimately resulting in the activation of different transcription factors such as Krüppel-like factor 2 (KLF2), necessary for cell survival, and monocyte chemoattractant protein 1 (MCP1), that regulates migration and infiltration of monocytes. Finally, shear stress is known to modulate the composition of the endothelial glycocalyx itself. For instance, low shear (4 dyn/cm^2^) upregulates syndecan-1 expression, while high shear (10–14 dyn/cm^2^) increases the expression of syndecan-4 (52, 53). Altogether, the glycocalyx, and syndecans in particular, seem to play an essential role in mechanotransduction of vascular shear stress forces to preserve diverse aspects of vascular physiology.

Physically, ECs control migration of molecules and cells across the vessel wall and prevent vascular leakage by creating a solid EC monolayer in which cells are interconnected through tight and adherens junctions (Figure 2C). Tight junctions form a paracellular barrier that regulates cellular permeability as well as an intramembrane barrier that restrains exchanges between the luminal and basolateral cell membrane (54). Instead, adherens junctions maintain tissue structure and, by transduction of signals, modulate cell specification and cell growth (55, 56). Although endothelial junctions are the major players in maintaining vascular integrity, the luminal glycocalyx layer is also involved by acting as a charge-selective barrier that restrains transport of molecules into the intra- and subcellular space. In line with this concept, proteins that display a similar charge interact with the glycocalyx in a similar manner *in vitro* and have similar entry rates to the intra- and subcellular space, despite their difference in size (e.g., albumin and fibrinogen) (57). The charge-based selectivity of glycocalyx *in vitro* is also demonstrated by Vink et al., who show that migration to the abluminal side of ECs is slower for anionic than for neutral dextran (58). Although *in vitro* damage of the glycocalyx influences EC permeability to proteins (23), *in vivo* settings revealed that vascular permeability is regulated by both the glycocalyx composition and the structure of EC junctions in a coordinated fashion (59). In fact, removal of HS from the endothelial glycocalyx layer reduces the expression of the gap junction protein connexin 43 by 30%, causing an increase in vascular permeability (60). This exemplifies how both the glycocalyx and the endothelial junctions are essential in maintaining vascular integrity and regulating vascular permeability.

In addition to the so far mentioned functions, the glycocalyx is also able to directly interact with different plasma proteins and circulating cells, regulating inflammation and coagulation (Figure 2D). The sulfated GAG side chain HS is the major player for these interactions and has been shown to express binding sites for different growth factors, cytokines, and chemokines (61). *Vice versa*, HS binding domains are represented in a wide range of plasma proteins. These domains are characterized by clusters of one to three basic amino acids interspaced with one or two non-basic residues such as glycine and serine (62). Examples of growth factors binding to HS include fibroblast growth factor (FGF), vascular endothelial growth factor (VEGF) (63), and granulocyte macrophage colony stimulating factor (GM-CSF) (64). Binding of these proteins to heparan sulfate proteoglycans (HSPGs) not only influences their stability, bioavailability and protects them from degradation, but also leads to an increase of their concentration on the cell surface, facilitating and amplifying intracellular signaling. For instance, IFNγ dimers and IL-8 bind to a small internal domain of HS rich in GlcA which leads to an increase in neutrophil migration (65–67). MCP1, Regulated on Activation Normal T-cell Expressed and Secreted (RANTES), and macrophage inflammatory peptides 1α and β (MIP-1α and MIP-1β, respectively) chemokines interact with the glycocalyx to generate the so called “chemokine-cloud,” a local concentration of chemokines within the glycocalyx layer (68). This facilitates leukocyte activation and amplifies pro-inflammatory signals (69).

Proteins that have binding sites for HS include ATIII (11, 13) which regulates hemostasis, superoxide and xanthine dismutase (SOD and XOD, respectively) (70, 71) that protects against oxidative stress, and complement fH (12) as well as C1 inhibitor which are involved in regulation of complement activation. The interaction of these proteins with HS potentiates their function. For example, interaction of fH with HSPGs enhances its capacity to bind and subsequently degrade C3b (72, 73). Given that HS is one of the main components shed during pathological conditions, it is more than plausible that the inflammatory and pro-coagulant conditions observed after glycocalyx shedding are due to loss of interaction of the glycocalyx with these regulatory plasma proteins. Analysis of such interactions would allow a better understanding of the pathophysiological mechanisms behind many inflammatory conditions.

In addition to interacting with plasma proteins the intact glycocalyx also acts as a barrier to leukocyte adhesion (Figure 2E) (21, 74). Rolling and adhesion of leukocytes to vessels is mediated by selectins and integrins expressed on activated ECs (75). The thickness of the intact glycocalyx, measuring up to one micrometer, exceeds the length of receptors involved in leukocyte adhesion which are only 20–40 nm long (76). Consistent with this, increased adhesion of leukocytes is observed only upon degradation of the glycocalyx (20).

On the other hand, HS has been shown to directly bind herpes simplex virus 1, human immunodeficiency virus 1 (HIV-1) (77, 78) and SARS-CoV-2, and facilitate entry into host epithelial cells and favor infection (79). Although HS is important for viral entry in epithelial cells, whether this is also true for ECs is still unknown.

In summary, an intact glycocalyx is essential to maintain vascular integrity and avoid coagulation dysregulations and leukocyte adhesion and subsequent transmigration into the surrounding tissue.

## Endothelial Glycocalyx Damage And Its Link To Risk Factors Of Atherosclerosis

Atherosclerosis is a chronic inflammatory process that induces cholesterol plaque formation within the artery wall. Accumulation of oxidized lipoproteins in the vessel wall first occurs at sites of endothelial dysfunction, characterized by increased permeability, impaired cellular communication and vessel tone, complement activation, leukocyte adhesion, platelet aggregation and alteration of the glycocalyx (80–83). During atherosclerosis, EC activation induces local inflammation and promotes monocyte migration to the intima of the vessel and their subsequent differentiation into macrophages. Oxidized lipoproteins are then taken up by macrophages giving rise to foam cells and finally leading to plaque formation (84). In addition, vessel injury induces a pro-thrombotic and anti-fibrinolytic EC phenotype predisposing the atherosclerotic lesion site to thrombosis and reducing NO production and release (85). This in turn impairs endothelial-dependent vasodilation further allowing accumulation of lipoproteins (86). Tsiantoulas et al. demonstrated that the intact HSPGs structures protect against atherosclerotic plaque development by interacting with A PRoliferation-Inducing Ligand (APRIL). Binding of APRIL to HSPGs reduces retention of lipoproteins and migration of macrophages to the vessel intima thereby limiting plaque formation (87). In contrast, reduced glycocalyx thickness is linked to increased lipid retention and plaque formation (88).

**Figure 3:**
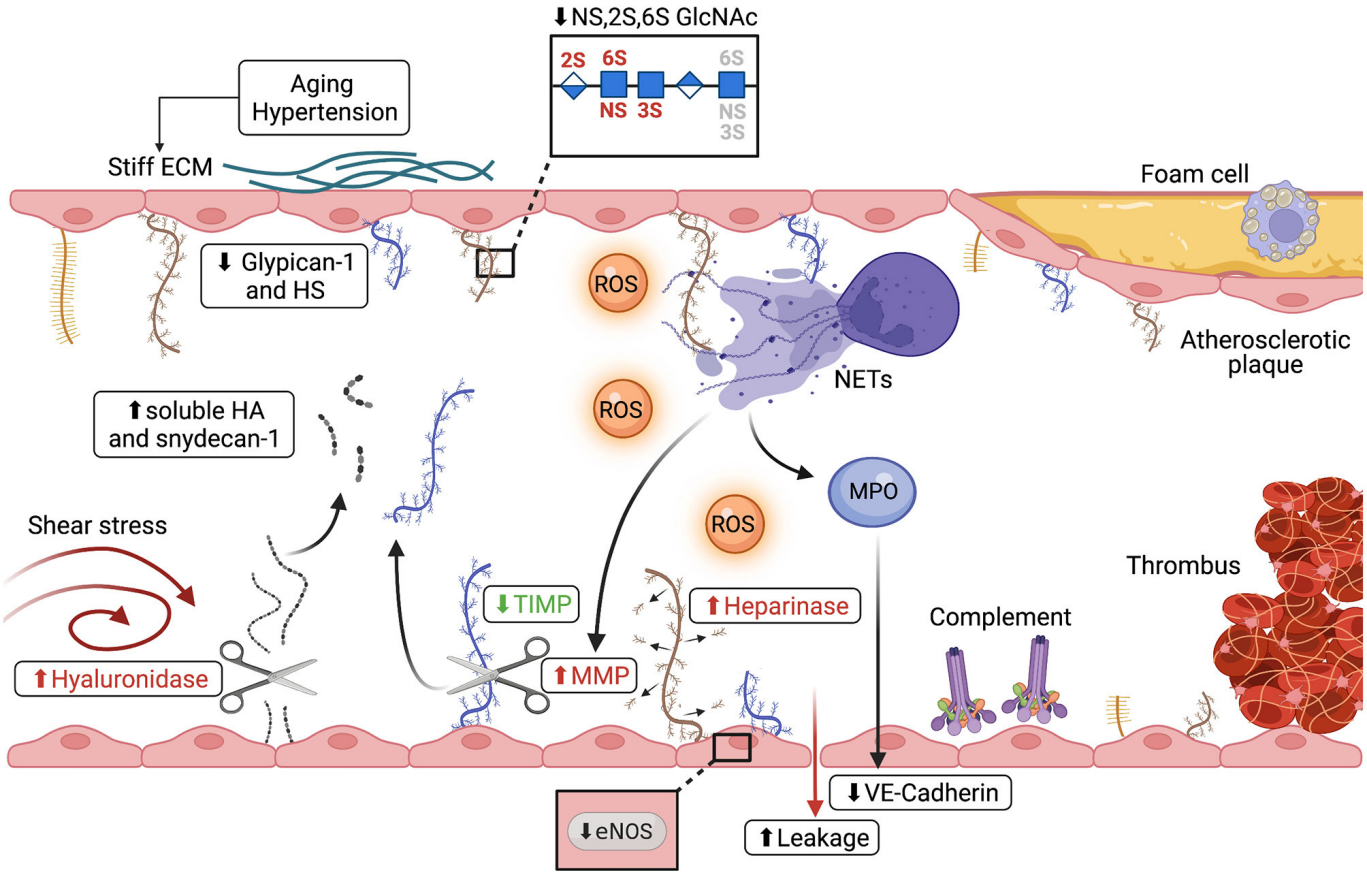
Alterations of the endothelial glycocalyx during thrombo-inflammatory conditions. Different mechanisms lead to shedding of the micro- and macrovascular glycocalyx in atherosclerosis and during ischemia/reperfusion injury (IRI). (I) Disturbed flow increases hyaluronidase expression leading to shedding of hyaluronan (shown in gray) and increased plasma syndecan-1 (shown in blue) and hyaluronic acid. (II) Hypertension, vascular stiffness and aging, all risk factors of cardiovascular disorders, cause thinning of the glycocalyx, evidenced by reduction of both glypican-1 and HS, as well as changes in the sulfation pattern of HS. (III) ROS (shown in orange), advanced glycation end products and sheddases all directly damage the endothelial glycocalyx and are linked to cardiovascular risk factors such as diabetes and obesity. (IV) Disruption of the glycocalyx is shown to be linked to reduced NO production and eNOS activity as well as activation of coagulation pathways and disturbed vasodilation. (V) Both complement deposition on the EC surface and neutrophil activation are associated with glycocalyx damage. Upon activation, neutrophils release neutrophil extracellular traps (NETs), decondensed chromatin decorated with neutrophil proteins. NETs can directly alter the glycocalyx or, through the release of MMPs and MPO (shown in purple), shed glycocalyx components and degrade junction proteins such as VE-Cadherin, leading to increased vascular leakage.

Most atherosclerotic lesions develop in vessel areas subjected to disturbed or reduced flow like bifurcations, curvatures and branching points (89). Disturbed or reduced flow profiles are known to damage the endothelial glycocalyx through the activity of sheddases, proteolytic enzymes that cleave glycocalyx components releasing soluble PGs or GAGs (Figure 3) (90). For example, low shear stress activates hyaluronidase, which induces shedding of hyaluronan from the glycocalyx leading to a thin and unstable glycocalyx layer (91). *In vivo* studies have confirmed the presence of a thin and disrupted glycocalyx layer in regions of vessels presenting disturbed flow. In the same areas atherosclerotic plaques were found (88). Interestingly, in patients with coronary heart disease, a thin endothelial glycocalyx layer was associated with a plasmatic increase of hyaluronic acid and syndecan-1 (Figure 3) (92, 93). This confirms that shedding of the glycocalyx and vascular dysfunction are key elements in atherosclerosis. In line with these findings, Nagy et al. proved that treatment of apolipoprotein E–deficient mice with 4-methylumbelliferone, an inhibitor of hyaluronan synthesis, damages the endothelial glycocalyx causing atherosclerosis (94). Loss of glycocalyx compromises the endothelial barrier functions resulting in an increase of vascular permeability. This is followed by cholesterol accumulation and macrophage infiltration of the vessel wall, which eventually leads to atherosclerosis (88, 95, 96). Although it appears to correlate with the formation of atherosclerotic plaques, glycocalyx integrity alone is still not considered a risk factor for the development of cardiovascular diseases in clinical settings (97). Nevertheless, cardiovascular risk factors for atherosclerosis such as hypertension, aging, diabetes, and obesity are known to damage the endothelial glycocalyx, underlining the importance of this endothelial structure in cardiovascular disorders. For instance, high sodium levels, typical of a high salt diet, reduce HS content on human ECs (98) and are associated with syndecan-1 shedding (99). In hypertensive patients, loss of glycocalyx integrity and reduced thickness is associated with increased vascular stiffness, which is an independent predictor of cardiovascular risk caused by degeneration of the extracellular matrix of elastic arteries (Figure 3) (100–102). Vascular stiffness is initiated by high blood pressure as well as vascular aging and it negatively affects the endothelial glycocalyx (103). In agreement, ECs cultured on stiff matrices that mimic rigid aged vessels, express low levels of the endothelial glycocalyx components HS, glypican-1 and hyaluronan compared to ECs cultured on a soft matrix (104). Furthermore, low levels of glypican-1 are found in a mouse model of age-mediated vascular stiffness and are associated with endothelial dysfunction. Knocking out glypican-1 in young mice results in a vascular phenotype typical of advanced age, characterized by arterial stiffness and endothelial dysfunction (105). Although endothelial glycocalyx damage is linked to vascular stiffness, the molecular mechanisms behind have not been elucidated so far.

Vascular aging, an important cardiovascular risk factor associated with increased vessel stifness, is characterized by microvascular dysfunction, impaired perfusion and reduced capillary density (106). In mice, glycocalyx thickness and integrity are reduced with age (107, 108). A reduction of the endothelial glycocalyx thickness of ~30% was observed also in humans with a mean age of 60 years compared to 30-year-old individuals (109). *In vitro*, HS is abundantly expressed on the surface of low-passage human umbilical vein ECs compared to cells at higher passages (110). However, this result needs a word of caution, as the study setup cannot discriminate between cell aging and phenotypic drift. Interestingly, not only the amount of HS on the surface of ECs is influenced by aging but also the HS sulfation pattern can differ in old compared to young individuals (Figure 3). Human blood outgrowth cells isolated from old individuals present with a lower amount of tri-sulfated NS, 2S, 6S N-acetylglucosamine as compared to cells from young individuals (111). This suggests that aging alters the fine structure of HS, which could lead to reduced plasma protein interaction and therefore interfere with hemostasis (112).

Other risk factors of atherosclerosis in which alterations in the endothelial glycocalyx have been observed are diabetes and obesity. Thinning of the glycocalyx and endothelial stiffness were detected in the aorta of diabetic mice, whereby the damage to the glycocalyx was associated with endothelial stiffening and a reduction in endothelial NO production (113). Consistent with this, hyperglycemia results in glycocalyx shedding, as evidenced by the plasma increase of hyaluronan and syndecan-4, endothelial dysfunction, activation of coagulation pathways and reduction of eNOS activity (114–116). The three main factors reported to result in glycocalyx degradation in diabetes are reactive oxygen species (ROS), advanced glycation end products and sheddase activation. Both ROS and advanced glycation end products depolymerize hyaluronan, causing a damage in the glycocalyx integrity, while sheddases, like hyaluronidase, heparinase, metalloproteinase and neuraminidase, cleave glycocalyx components (Figure 3) (117). Notably, all these factors are increased in plasma of diabetic patients, which indicates a strong correlation between glycocalyx damage and insulin resistance (118–120).

Obesity is also known to induce endothelial dysfunction and, as for diabetes, it can manifest as loss of glycocalyx barrier properties and reduced NO availability (121, 122). The dilatory response of arteries to flow is impaired during obesity. This loss of flow-induced vasodilation is due to a decrease in NO production (123). Recent studies have shown that the activity of the flow sensitive K+ channel Kir, responsible for vasodilation, is highly dependent on HS. Heparinase treatment of freshly isolated ECs from obese mice reduces Kir activity. Interestingly, impairment of flow-mediated vasodilation in obesity differs between vascular beds. Endothelial dysfunction is observed in visceral adipose arteries but not in subcutaneous adipose arteries, where Kir activity, glycocalyx integrity and normal vasodilation are maintained (124). In contrast, *in vivo* data revealed that a thicker glycocalyx in the brain vasculature of obese mice is protective against inflammation during critical pathological conditions (125). This so called “obesity paradox” suggest that the glycocalyx might be structurally and functionally different in distinct organs, thus revealing an unexpected level of structural complexity.

## Shedding Of Endothelial Glycocalyx During Ischemia/Reperfusion Injury

Ischemia/reperfusion injury (IRI) refers to the tissue damage sustained once blood flow is re-established after an ischemic period and can occur under different circumstances such as myocardial infarction with thrombotic vessel occlusion, heart surgery, transplantation, and ischemic stroke. Common to these circumstances are (I) tissue damage due to ROS produced upon reperfusion (81) (II) mitochondrial dysfunction with opening of the mitochondrial transition pore and loss of ATP production (126) (III) activation of the complement system (127, 128), and (IV) endothelial dysfunction (129). Glycocalyx shedding is recently emerging as an additional common event during IRI. Animal models of cardiac IRI have shown that the thickness of the glycocalyx is reduced as early as 5 min after reperfusion and that its shedding leads to decreased endothelium dependent vasodilation mediated by NO (130, 131). Early shedding of syndecan-1 and HS was also observed during reperfusion in patients undergoing cardiac surgery (132).

ROS are known to be key players in glycocalyx shedding/damage during IRI. Administration of the anti-oxidative reagent superoxide dismutase (SOD) maintains glycocalyx integrity and protects microvessels from IR damage (133). In agreement with this, patients with acute coronary syndrome, survivors of cardiac arrest or patients undergoing coronary bypass all present increased blood levels of circulating glycocalyx components such as syndecan-1 (92) and HS (15, 134). This has also been confirmed in ischemic stroke patients, where multiple soluble glycocalyx components, including 3 different GAGs (HS, keratan sulfate, chondroitin sulfate) and 3 different PGs (CD44, syndecan-2 and -3), were increased in the plasma of patients 1 week after the initial incident (135). Taken together, these studies indicate that mainly the core PG syndecan-1 and the GAG side chain HS are disrupted in IRI. In particular, HS shedding during IRI could explain some of the observed pathological vessel changes like increased permeability, complement activation, thrombosis and leukocyte infiltration toward the damaged tissue. In fact, one of the earliest inflammatory responses during cardiac IRI, next to endothelial dysfunction, is the activation of the complement system and the interaction of innate immune cells, such as neutrophils, with the vessel wall (136).

Different animal studies have shown the involvement of the complement system in IRI (137). Deposition of the complement components C3d and C5b-9 was seen in reperfused hearts of myocardial infarction patients (138) and associated with an increase in shedding of syndecan-1, a core protein of the endothelial glycocalyx (139, 140). Complement inhibition, by administration of a membrane-targeted molecule derived from complement receptor 1 (141) or by administration of dextran sulfate, was shown to be beneficial in experimental myocardial infarction as well as in cardiac transplantation (142). Dextran sulfate binding to the damaged myocardial blood vessels correlated with reduced vascular staining for HSPGs, suggesting a replenishment of the shed glycocalyx by dextran sulfate (143). Although complement deposition is correlated with loss of glycocalyx integrity, the exact link between the complement system and degradation of the glycocalyx in IRI is not very well-understood. On one hand, degradation of the glycocalyx might cause loss of interaction of complement regulatory proteins present in plasma such as fH or C1-inhibitor with the endothelial glycocalyx, resulting in increased complement deposition and tissue injury (144, 145). On the other hand, IRI might cause the expression of neoantigens on the EC surface (81). Binding of naturally occurring IgM antibodies to the neoantigens can then in turn lead to complement activation and tissue injury (146). The endothelial glycocalyx not only interacts with regulatory plasma proteins but also shields off cell surface adhesion molecules thereby limiting their interaction with immune cells. Shedding of the glycocalyx during both myocardial infarction and stroke has been shown to contribute to vascular edema as well as neutrophil and platelet adhesion to the vessel wall (147). Figure 3 illustrates how during neutrophil-mediated immune response, shedding of glycocalyx constituents can occur via enzymatic digestion by metalloproteases (MPOs) and hyaluronidase, or non-enzymatic degradation through oxidative stress. Other than degrading the glycocalyx, neutrophil enzymes such as MPOs, elastases, and cathepsins, can also cleave endothelial cell-cell junctions, in particular VE-Cadherin, leading to impaired junction integrity and vascular leakage (148). During myocardial infarction, stroke, and peripheral vascular disease, activated neutrophils also release neutrophil extracellular traps (NETs), web-like structures of decondensed chromatin covered with histones and cytoplasmic and granular proteins (149). It is well-accepted that the extracellular histones released during NET formation are highly cytotoxic for ECs and interact with the endothelial glycocalyx causing microvascular leakage and barrier dysfunction (150, 151). In fact, incubation of microvascular ECs *in vitro* with calf thymus histones results in EC death. Interestingly, this cytotoxicity is prevented by addition of negatively charged heparan sulfate tetra- or decasaccharides (152). Nucleosomes and histone 4 have been also detected in perfusates of isolated rat hearts subjected to ischemia reperfusion. Higher histone levels correlated to bigger infarct size (153).

Both neutrophil activation and complement deposition are considered key players in loss of endothelial glycocalyx integrity during IRI (Figure 3). In fact, knock-out of the complement receptor 5a in a mouse model of myocardial infarction resulted in reduction of neutrophil transmigration to post ischemic myocardium and diminished the expression of the matrix metalloproteinase 9, a sheddase of the endothelial glycocalyx (154). Both sheddases and sulfatases alter the endothelial glycocalyx by respectively removing entire GAG side chains and PGs or by changing the sulfation pattern of the GAG side chains. Many different sheddases are upregulated during IRI and are shown to be responsible for endothelial glycocalyx degradation. For instance, tryptase β (155), heparinase (156, 157) and atrial natriuretic peptide (ANP) (158) lead to an increase of soluble syndecan-1 (159). Similarly, the activity and release of matrix metalloproteinases (MMPs), which cleave entire PGs, is also upregulated during IRI. Cardiomyocytes and neutrophils have been identified as possible sources for MMPs during IRI (160, 161). Although ECs also produce MMPs, it is not clear whether MMPs of endothelial origin contribute to IRI (162). Shedding of syndecan under ischemic conditions is not only caused by upregulation of MMPs but is also due to downregulation of their natural inhibitors, the tissue inhibitors of metalloproteinases (TIMPs) (163–165). This indicates that IRI modulates not only the release of sheddases but also regulates their enzymatic activity. Other than syndecans, also removal of hyaluronan from the glycocalyx has been observed during ischemic stroke indicating that the sheddase hyaluronidase is involved in IRI (166, 167).

Finally, sulfatases can be upregulated during IRI. However, differently to sheddases, sulfatases seem to have a protective role. In a mouse model of myocardial infarction, the increase of sulfatase-1 and−2 was associated with a decrease in HS 6-O sulfation. This led to a reduction in the interaction of the glycocalyx with VEGF in the infarcted zone enhancing its bioavailability and increasing ischemic tissue repair (168). It is currently unknown whether upregulation or downregulation of sulfatases can have a detrimental effect on IRI.

## Soluble Endothelial Glycocalyx Components As Regulators Of Inflammation

The endothelial glycocalyx regulates inflammation through different mechanisms: (I) it shields off integrins and other co-stimulatory molecules inhibiting binding of leukocytes to the endothelial surface, impeding antigen presentation and T cell activation, (II) it creates a local chemokine gradient that favors leukocyte activation (69, 169) (III) it regulates the activity of chemokines, cytokines and growth factors by protecting them from enzymatic degradation (68) and (IV) it binds regulatory proteins such as factor H or, in a soluble form, mannan-binding lectin serine protease 2 (MASP-2), thereby mediating complement inhibition (170).

**Figure 4:**
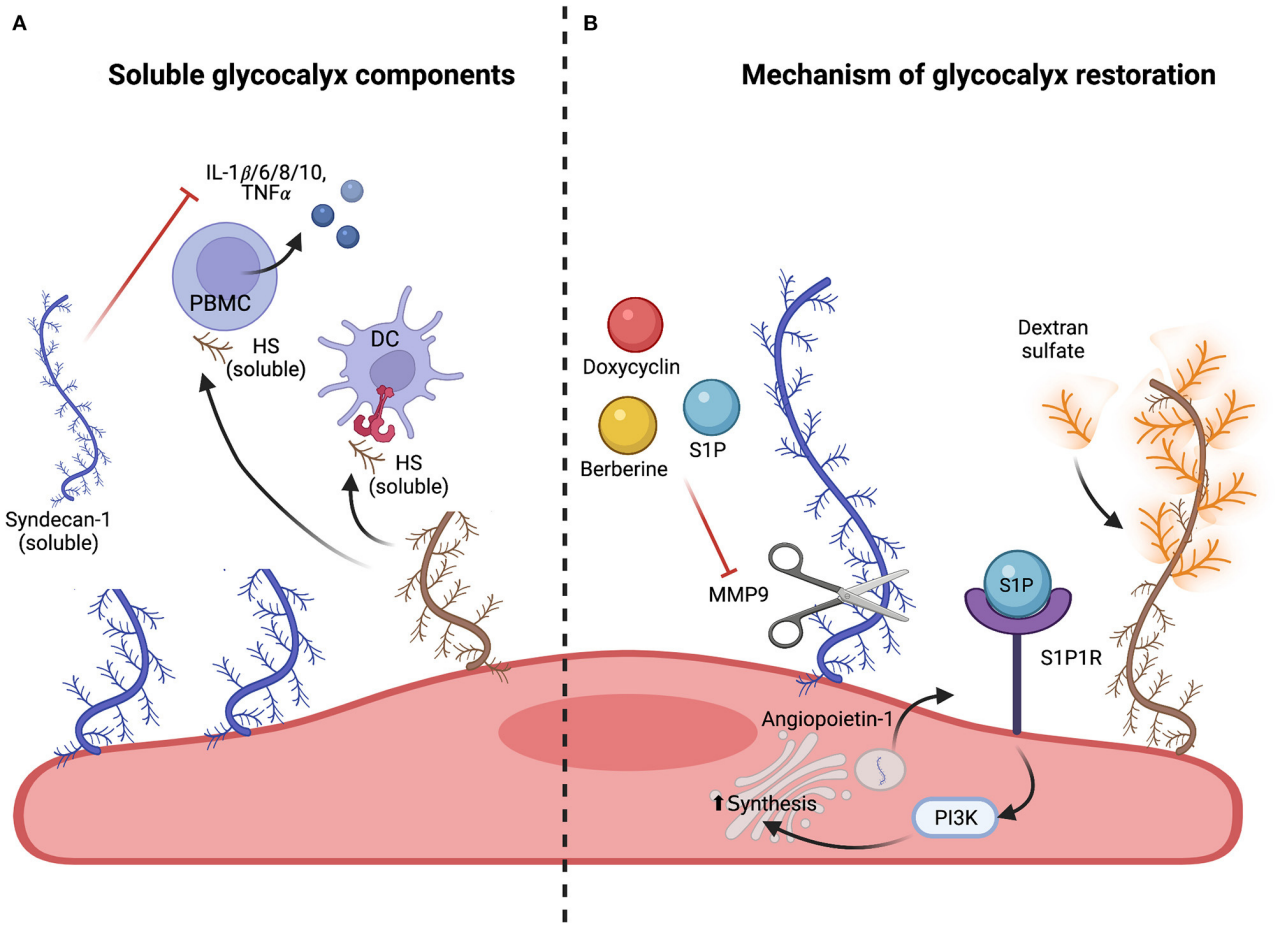
Soluble glycocalyx components and mechanism of glycocalyx restoration. **(A)** Effect of soluble glycocalyx components. Released soluble glycocalyx components such as HS (brown), syndecan-1 and −3 (blue) can propagate inflammatory responses by activating peripheral blood mononuclear cells (PBMCs) and dendritic cells (DCs) *via* TLR4 signaling. This leads to release of pro-inflammatory cytokines and DC maturation. However, soluble syndecan-1 (blue) has also been shown to reduce inflammation by directly inhibiting cytokine and chemokine release and blocking leukocyte adhesion. **(B)** Protection and restoration of the endothelial glycocalyx. Maintenance of glycocalyx integrity can be achieved by inhibition of sheddases. Doxycycline (shown in red), berberine (shown in yellow) or S1P (shown in blue) have been shown to directly inhibit matrix metalloproteinase 9 (MMP9) and therefore impede glycocalyx shedding. Regeneration of the glycocalyx can be achieved by upregulating the expression and extravasation of glycocalyx components such as syndecans with S1P and angiopoietin-1 or by replacing shed components such as HS with structurally similar agents such as dextran sulfate (shown in orange), a highly branched polysaccharide.

Recent studies have shown that upon shedding of the glycocalyx, soluble HS propagates inflammation by directly interacting with toll-like receptors (TLRs). *In vitro* experiments proved that soluble HS fragments can stimulate the release of pro-inflammatory cytokines such as IL-1β, IL-6, IL-8, IL-10, and TNFα from human peripheral blood mononuclear cells (18). Additionally, both HS and hyaluronan induce dendritic cell maturation by TLR4 signaling (Figure 4A) (171). In agreement, mutation of TLR4 or incubation of dendritic cells with the TLR4 antagonist s-DPLA inhibited the upregulation of the co-stimulatory molecules CD86 and CD40 on the cell surface (172). The overall impact of soluble HS on vascular physiology remains largely debated. On one hand, stimulation of freshly isolated cardiac fibroblasts with soluble HS increases, through TLR4 signaling, the expression of ICAM-1 and VCAM-1 on the cell surface, favoring the adhesion of spleen mononuclear cells and bone marrow granulocytes. This promotes cardiac fibroblast differentiation into myofibroblasts. On the other hand, treatment of cardiac fibroblasts with HS was able to reduce alpha smooth muscle actin expression, impeding the differentiation into myofibroblast and protecting against a pro-fibrotic phenotype. This suggests that soluble HS can be both protective and deleterious (173).

Although it is well-accepted that HS binds TLR4, the molecular mechanism of action of soluble HS is still not fully understood. Studies suggest that HS signaling through TLR4 activates a different NFκB pathway with respect to the response induced by the TLR4 ligand lipopolysaccharide (LPS). Indeed, TLR4 activation by soluble HS triggers a slower translocation of NFκB to the nucleus when compared to activation by LPS, and a more sustained increase of intracellular levels of calcium in macrophages (174). Whether this gives rise to different immune responses has not been yet investigated. Also, it is still unclear whether the main source of soluble HS derives from shedding of endothelial glycocalyx or from heparinase degradation of the extracellular matrix (172). Similarly, soluble syndecan-4 propagates the inflammatory response by upregulating ICAM1, VCAM1, IL-1β, and TNFα expression on cardiomyocytes increasing inflammatory cell recruitment (175).

Interestingly, soluble glycocalyx components not only propagate inflammation, but can also downregulate inflammatory signaling. For example, soluble HS fragments can directly inhibit cytokines such as IL-10 and interact with leukocytes, blocking their adhesion to the EC surface (Figure 4A) (176, 177). Addition of syndecan-1, which is rich in HS, was shown to directly inhibit the accumulation of CXC chemokines, thereby reducing neutrophil accumulation in various organs in a murine endotoxemia model (178). Along the same line of research, syndecan-1 was shown to block the expression of IL-1β, IL-6, and TNFα and the activity of pro-inflammatory chemokines CCL7, CCL11, and CCL17 (179, 180). Shed syndecan-3 also binds inflammatory chemokines like CCL2, CCL7, and CXCL8 and inhibits leukocyte migration in a mouse model of rheumatoid arthritis (181). Although syndecan-3 expression increases during myocardial infarction, its role in cardiovascular disorders is unknown (182).

Soluble components of the EG have the potential to both propagate and inhibit inflammation. The mechanism leading toward one direction rather than the other is still unknown and should be investigated.

## Protection And Regeneration Of The Endothelial Glycocalyx

A damaged glycocalyx can regenerate over time. Although *in vivo* experiments have shown that 7 days are sufficient to reinstate the endothelial glycocalyx layer following enzymatic digestion, the mechanism behind and whether the restored glycocalyx is similar in its composition to the original has not been investigated so far (183). Nevertheless, various preventive and restorative approaches, aimed to re-establish the physiological glycocalyx function, have been suggested as potential therapies for several inflammatory disorders.

Different molecules can prevent endothelial glycocalyx degradation (Table 1). For example, angiopoietin-1 (184), hydrocortisone (189) ATIII (188), and SOD (133) maintain vascular integrity and prevent endothelial dysfunction during IRI by impeding the degradation of the glycocalyx components syndecan-1, HS and hyaluronic acid and have therefore been proposed as potential therapeutic candidates. However, how these molecules support endothelial glycocalyx integrity is still unclear. In Figure 4B, the two different proposed mechanisms of action are shown: angiopoietin-1 has a fast-acting effect in preventing glycocalyx shedding, suggesting that it protects the glycocalyx not by *de novo* synthesis of its components but rather by translocation of pre-formed GAG components from the Golgi apparatus to the cell surface. In fact, inhibiting the translocation of vesicles abolished the protective effect of angiopoietin-1. The second mechanism, proposed for hydrocortisone and ATIII, is a direct inhibition of sheddases which prevents glycocalyx degradation. One of the most abundant sheddases responsible for glycocalyx damage is the matrix-metalloproteinase-9 (MMP9). MMP9 belongs to a family of zinc-dependent endopeptidases implicated in both physiological and pathophysiological tissue remodeling. MMP9 is regulated transcriptionally by NFκB and once synthesized, its enzymatic activity is modulated by endogenous TIMPs (201). Berberine (187), doxycycline (185), and sphingosine-1 phosphate (S1P) (186) alleviate endothelial glycocalyx degradation by inhibiting MMP9 (Table 1, Figure 4B). Interestingly, S1P not only avoids glycocalyx shedding by directly interfering with MMP activity, but it also restores the glycocalyx once damaged. In fact, as depicted in Figure 4B, S1P interacts with its receptor S1P1R expressed on ECs and increases the synthesis of syndecan-1 and HS *via* intracellular phosphatidyl inositol-3 kinase (PI3K) signaling (60, 202).

**Table 1:** Protection and regeneration of the endothelial glycocalyx.

	**Molecule**	**Pathology**	**Mechanism**	**References**
Protection of the endothelial glycocalyx	Angiopoietin-1	Microvascular inflammation	Translocation of intracellular vesicles containing glycocalyx components to the cell surface	(184)
	Doxycycline		MMP inhibition (hypothesis)	(185)
	Sphingosine-1 phosphate		Syndecan-1 shedding inhibition	(186)
	Berberine	Lipopolysaccharide-induced acute respiratory distress syndrome (ARDS)	ROS and MMP inhibition	(187)
	ATIII	IRI	MMP inhibition (hypothesis)	(188)
	Hydrocortisone		Prevention of mast cell degranulation and MMP release	(189)
	SOD		Inhibit ROS mediated glycocalyx degradation	(133)
	Sevoflurane	Oxidative stress	Increased glycocalyx synthesis	(190)
	Sulodexide	Diabetes	Heparanase-1 inhibition	(191)
Regeneration of the endothelial glycocalyx	Sphingosine-1 phosphate	Enzymatic removal of the glycocalyx	Not known	(60)
	Empaglifozin		Not known	(192)
	Adjunct drugs: adenosine-lidocaine-magnesium (ALM), beta-hydroxybutyrate plus melatonin (BHB/M), and poloxamer 188 (P-188)	Hemorrhagic shock	Counteraction of ROS toxicity	(193)
	Restoration with plasma		Release of pre-formed intracellular syndecan-1	(194)
	Hydroxyethyl starch resuscitation		Downregulation of heparinase, hyaluronidase and neuraminidase	(195)
	Secreted protein acidic and rich in cysteine (SPARC)	Myocarditis	Not known	(196)
	Dextran sulfate	Cardiac xenotransplantation	“Repair coat,” local replacement of shed HS	(143, 197, 198)
	Sulfated tyrosine	Xenotransplantation model		(199)
	MMP inhibitor	Diabetes	MMP inhibition	(200)

*Overview of the different approaches to protect (upper part) or restore (lower part) the endothelial glycocalyx including proposed mechanism of action*.

Different studies have shown that also adjunct drugs (193), restoration with plasma (194), heparin (203, 204), sulodexide (191), and hydroxyethyl starch downregulate heparinase, hyaluronidase and neuraminidase (195) during inflammation or sepsis and therefore prevent endothelial dysfunction by preserving glycocalyx integrity (Table 1). Whether these approaches are beneficial also for cardiovascular disorders is not known. Also, the respective mechanism of action is still unclear. Dextran sulfate, a highly branched polysaccharide resembling HS, has also been recommended as a potential EC protectant. It was suggested that dextran sulfate creates a “repair coat” that mimics the missing endothelial glycocalyx layer and thereby inhibits complement deposition during acute vascular rejection (Table 1, Figure 4B) (197, 198, 205). Further studies are needed to confirm such a hypothesis.

Upregulation of glycocalyx synthesis genes could be a secondary approach for restoration of the endothelial glycocalyx during disease. However, little is known on the beneficial effects of induction of glycocalyx gene expression in damaged cells. It was shown that the anesthetic sevoflurane increases sialyltransferase expression during oxidative stress thereby promoting endothelial glycocalyx restoration and vasodilation (Table 1) (190). This suggests that other interventions leading to an increase of expression of glycocalyx synthesis genes could be helpful and open new avenues of research in the field of glycocalyx restoration.

Although shedding of the endothelial glycocalyx has been related to various cardiovascular and inflammatory conditions, a clear understanding of the mechanisms involved in glycocalyx shedding is still missing. Nonetheless, glycocalyx disruption occurs already early during disease development, suggesting that glycocalyx components could be potentially important, early biomarkers of disease. Given that prevention of glycocalyx shedding can be achieved, recognizing glycocalyx damage and inhibiting shedding might be beneficial in slowing down or even preventing certain cardiovascular conditions. Further research should focus on understanding the molecular mechanisms behind glycocalyx injury and uncover new therapeutic options for the protection or restoration of the endothelial glycocalyx in cardiovascular disorders.

## Author Contributions

AM wrote the manuscript. NS and RR revised the manuscript. NS supervised the review. All authors approved the submitted version.

## Funding

This study was supported by Swiss National Science Foundation, grant no. 310030_182264.

## Conflict of Interest

The authors declare that the research was conducted in the absence of any commercial or financial relationships that could be construed as a potential conflict of interest.

## Publisher's Note

All claims expressed in this article are solely those of the authors and do not necessarily represent those of their affiliated organizations, or those of the publisher, the editors and the reviewers. Any product that may be evaluated in this article, or claim that may be made by its manufacturer, is not guaranteed or endorsed by the publisher.
